# Is the Apert foot an overlooked aspect of this rare genetic disease? Clinical findings and treatment options for foot deformities in Apert syndrome

**DOI:** 10.1186/s12891-020-03812-2

**Published:** 2020-11-28

**Authors:** Alexandra Stauffer, Sebastian Farr

**Affiliations:** Department of Pediatric Orthopaedics and Foot and Ankle Surgery, Orthopedic Hospital Speising, Speisingerstrasse 109, 1130 Vienna, Austria

**Keywords:** Apert syndrome, Foot deformity, Treatment options

## Abstract

**Background:**

Apert syndrome is characterised by the presence of craniosynostosis, midface retrusion and syndactyly of hands and feet, thus, synonymously referred to as acrocephalosyndactyly type I. Considering these multidisciplinary issues, frequently requiring surgical interventions at an early age, deformities of the feet have often been neglected and seem to be underestimated in the management of Apert syndrome. Typical Apert foot features range from complete fusion of the toes and a central nail mass to syndactyly of the second to fifth toe with a medially deviated great toe; however, no clear treatment algorithms were presented so far. This article reviews the current existing literature regarding the treatment approach of foot deformities in Apert syndrome.

**State-of-the-art topic review:**

Overall, the main focus in the literature seems to be on the surgical approach to syndactyly separation of the toes and the management of the great toe deformity (hallux varus). Although the functional benefit of syndactyly separation in the foot has yet to be determined, some authors perform syndactyly separation usually in a staged procedure. Realignment of the great toe and first ray can be performed by multiple means including but not limited to second ray deletion, resection of the proximal phalanx delta bone on one side, corrective open wedge osteotomy, osteotomy of the osseous fusion between metatarsals I and II, and metatarsal I lengthening using gradual osteodistraction. Tarsal fusions and other anatomical variants may be present and have to be corrected on an individual basis. Shoe fitting problems are frequently mentioned as indication for surgery while insole support may be helpful to alleviate abnormal plantar pressures.

**Conclusion:**

There is a particular need for multicenter studies to better elaborate surgical indications and treatment plans for this rare entity. Plantar pressure measurements using pedobarography should be enforced in order to document the biomechanical foot development and abnormalities during growth, and to help with indication setting. Treatment options may include conservative means (i.e. insoles, orthopedic shoes) or surgery to improve biomechanics and normalize plantar pressures.

**Level of evidence:**

Level V.

## Background

Apert syndrome [1] is characterised by the presence of multisuture craniosynostosis, midface retrusion and syndactyly of hands and feet, thus, synonymously referred to as acrocephalosyndactyly type I. This disorder occurs in 6 to 15.5 out of 1 million livebirths depending on the study cited, with an almost equally affected sex ratio [2–4]. The prevalence may be higher amongst children born to fathers with advanced paternal age, as the greatest variant of sperm mutations occurs in fathers above 60 years of age. Furthermore, there seems to be a paternal-age effect, as children with Apert syndrome are born to fathers who exhibit an increased frequency of mutant sperm at an earlier age [5]. Apert syndrome results from mutations localised on the FGFR2 caused by specific missense substitutions involving adjacent amino acids, thus resulting in two genotype-phenotypes consisting of Pro253Arg, causing more significant hand and foot involvement, and Ser252Trp [6].

Diagnosis is established in a patient clinically or by genetic testing resulting in the identification of heterozygous pathogenic variant of the FGFR2 and the presences of clinical features consistent with Apert syndrome. Almost all patients present with craniosynostosis commonly involving bilateral coronal sutures, however, a majority also displays a progressive fusion of the sagittal and lambdoid sutures. Unlike other acrocephalosyndactyly syndromes, the midface in Apert syndrome is underdeveloped, contributing to the development of prominent eyes with downslanting palpebral fissures, as well as retruded midface, thus leading to a shorter maxillary bone. A multitude of other abnormalities have been described in Apert syndrome affecting inner organs and other structures, such as spinal fusions, palatal and/or dental abnormalities, ocular abnormalities, hearing loss or inner ear anomalies, as well as multilevel airway obstructions, cardiac abnormalities, gastrointestinal issues, abnormalities of the genitourinary tract and various neurologic findings [7].

Considering these multidisciplinary issues, frequently requiring surgical interventions at an early age, deformities of the feet have been forced into the background and seem to be often overlooked in the management of Apert syndrome. Although these typical clinical appearances in Apert syndrome regarding feet have been described in multiple case reports ranging from complete fusion of the toes and a central nail mass [8] to syndactyly of the second to fifth toe with a medially deviated great toe [9], no treatment algorithms were presented so far. The purpose of this state-of-the-art topic article is to review the current existing literature regarding the approach to the treatment of foot deformities in Apert syndrome.

## The Apert foot and its treatment

Overall, the main focus in the literature seems to be on the surgical approach to 1) syndactyly separation of the toes and 2) the management of the great toe deformity. The following part is divided into sections addressing these main topics, as well as joint fusions and anatomical variants.

### Syndactyly of the toes

**Fig. 1:**
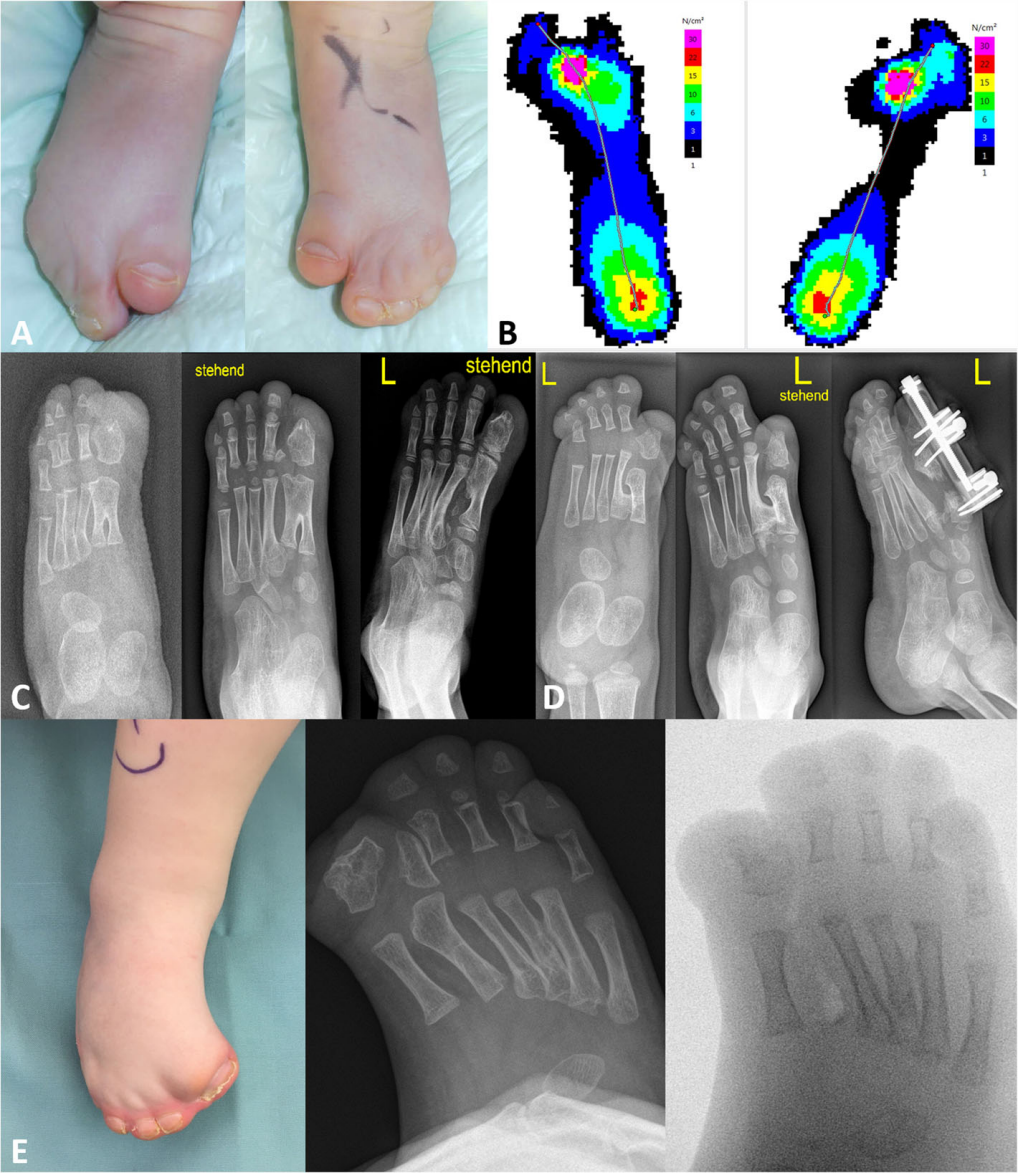
Left and right foot of a 3-year old boy with Apert’s syndrome showing complex syndactyly of toes 2 to 4, as well as a shortened and medially deviating great toe as a typical deformity (**a**). Pedobarography of a 4-year-old boy with Apert’s syndrome. Pressure distribution shows a shift laterally, with the maximum weight bearing along the metatarsals 3 and 4 on the left foot (84,7 N/cm^3^). The right foot shows a similar shift of the pressure points, the maximum is on the third metatarsal with a smaller weight bearing area (95 N/cm^3^) (**b**). Images of a 6-month old child with an interposed additional ray are shown. At the age of 4 years one can depict the calcaneocuboidal fusion, a synchondrosis between the metatarsal III base and the distal tarsals as well as an ossification between the great toe and the second toe. The final image after surgery 2 years postoperatively is shown. The additional full ray was resected, and a metatarsal II elevation osteotomy was performed to reduce the plantar pressure below it (**c**). Images of another case, 1 year and then 5 years of age, reveals an osseous bridge between metatarsal I and II, marked shortening of the first ray and mild hallux varus. A gradual lengthening procedure was performed to elongate the shortened ray (**d**). Images of a case (Blauth type III) with complete syndactylies I-V and forefoot adductus are shown. The radiographs show marked forefoot adduction partially caused by an interposed supernumerary dysplastic ray. Following resection of this ray the forefoot position and shape was markedly improved (**e**)

Three types of syndactyly of the toes have been described by Blauth et al. [10]. In a study which included 37 feet, Cohen et al. reported the predominant type being type III which was present in 54% of their patients. In type I, a fusion of the second, third and fourth toe is present, with one and five being separate. Other findings include a broad great toe which may shorten over time and deviate medially. According to Cohen et al. this type occurs in 27% of analysed cases. Type II (19% of cases) consists of a fusion which involves all toes except for the great toe, resulting in a supination of the forefoot (Fig. 1a). The position of the great toe is in varus and dorsiflexion. A fusion of all toes with a possibly hyperextended great toe is present in type III [11].

Fearon et al. proposed a two-stage approach as initial treatment of syndactyly release. Both stages consist of syndactyly release, however, stage one is performed at around 12 months of age, followed by the second stage 3 months later. The digits are separated distally to proximally through Z-plasties, and all distal bony fusions are divided to create normal phalangeal width, the neurovascular bundles are carefully separated, the skin is approximated and full-thickness skin grafts, which were harvested from the groin, transplanted into open spaces along the phalanges if required. The late stage is performed between the ages of 9 to 12 if necessary and includes osteotomies to create a permanent angulation of the digits in order to achieve a better functional position. This pertains to symptomatic foot disorders, which can be corrected by ostectomy of either the first or fifth metatarsal head to narrow the affected area. Elevation of the second metatarsal head through osteotomy and reangulation may be necessary if it becomes overly prominent on the plantar surface [12].

Upton et al. suggested separation of the toes at an early age, as interdigital skin grafts require immobilization to heal. They noticed that the skin transplants did not heal well for active children, regardless of whether or not they were treated with casts or splints. Furthermore, they noticed that the severity of the foot deformity did not correlate with the extent of hand deformity, especially in patients with type II and especially type III hands [13].

As a result of an osseous syndactyly subsequently changes in the nailbed may occur, the most frequent change being an acneiform eruption. Other occurrences include complete nail fusion with a single wide nail, central indentations, short nails with absent lunulae, and micronychia [14]. Narrowing of the nail may be achieved through fulguration of the lateral germinal matrix with electrocautery [12]. Other cutaneous changes include the formation of painful calluses due to altered weight bearing mechanics, especially over the protuberant second and third metatarsal heads [15]. Osteotomies of the metatarsals for stress relief have been reported in literature [16].

### Deformities of the great toe

Deformities of the great toe in patients with Apert syndrome were described by Dell et al. in 1981 [17]. Their study included case reports of three children aged between 2 and 3 years of age who showed significant difficulty in shoe fitting which lead to the decision to operate rather than to accept the aesthetic appearance of the foot. The first case presented with complex syndactylies of all toes and the presences of five toe-nails. Weight bearing took place on the lateral four rays (Fig. 1b), as plantar pressure of the first ray decreases with shortening [17]. Radiographs revealed a complex syndactyly between the first and second metatarsal with the presence of osseous fusion, as well as a delta phalanx proximally and an abnormally large and misshapen distal phalanx of the great toe. In order to correct this deformity, realignment of the great toe was performed by means of second ray deletion, resection of the delta phalanx on one side and corrective open wedge osteotomy on the other, furthermore, osteotomy of the osseous fusion and narrowing of the distal phalanx by longitudinal osteotomies were performed, as well as fixation of realignment of the great toe using K-wire, which were placed across the metatarsal-tarsal joints, and Z-plasty.

The second case presented with bilateral hallux varus deformities with shortening of the great toe, as well as syndactyly of the second, third and fourth toe, respectively. Radiographic findings included duplication of the proximal phalanx and a widened distal phalanx in the great toe. Measures to narrow the feet were undertaken, which included resection of the great toe and transposition of the second ray medially.

The last case presented with syndactyly of all toes, bilateral fusion of the first and second metatarsal, duplication (Fig. 1c) of the distal phalanx of both second toes and a medial angulation of the great toe. However, no shoe fitting problems were described by the parents, thus, no surgical intervention took place [18].

While Dell et al.’s approach to the realignment and correction of the great toe included a deletion of the second ray, other authors developed a different technique which allows for the preservation of the second ray. In a patient with Apert syndrome who presented with synostosis between the first and second ray, bilateral hallux varus, as well as metatarsus adductus of the second, third and fourth ray, Blauth et al. proposed a different surgical approach. They performed a resection of the osseous fusion between the first and second metatarsal, carried out an osteotomy of the second and third metatarsal, completed the correction of hallux varus by means of resecting the proximal rudimentary bone fragment and accomplished the realignment of the great toe using K-wire as a treatment option [19].

As the great toe shortens and broadens over time compared to the rest of the foot and the presence of a delta phalanx may lead to medial deviation and varus angulation, thus surgical measures such as correction of brachymetatarsia and medial angulation may be necessary. Calis et al. reviewed the long-term outcome of distraction osteogenesis (Fig. 1d) for this deformity in 14 Apert feet. They performed an osteotomy of the first metatarsal sparing the epiphyseal plate and inserted two pins proximal and distal to the osteotomy site, which were then connected to an external distractor device. Distraction protocol consisted of initiation 5 days postoperatively and a distraction rate of 0.5 mm per day. The mean distraction period observed was 12.6 ± 1.7 days, after a mean consolidation period of 62.6 ± 5.8 days, distractors were removed. Even though they observed complications such as pin loosening, pin infection and early union requiring revision surgery, Calis et al. reported a correction of brachymetatarsia long-term in all patients [20]. To this day, some surgical measures as performed by the authors mentioned above are still considered when formulating an operative plan for patients with deformities of the feet in Apert syndrome (Fig. 1e).

### Joint fusions and other anatomical variants

Schauerte et al. reported on bony fusions in 1966, observing that a fusion between the tarsal bones was the most constant finding in patients with Apert syndrome. They reported on cases showing progressive synostosis of the tarsal bones, in particular a fusion of the calcaneus and the cuboid, as well as fusions of the 3rd metatarsal with the lateral cuneiform or the navicular with the medial cuneiform [21].

Upton et al. proposed a classification into two groups, depending on the extent of fusion between the first two rays. In type I feet, the separation between the cuneiform bones and the first metatarsal are present at birth. Type II feet displayed more severe skeletal deformities, as fusions between the cuneiform bones and the navicular were present, as well as complete or partial fusions with the second metatarsal [22].

Collins et al. used computer tomography (CT) scans to define the spatial dysmorphology of the foot in a series of case reports of patients with Apert syndrome. They observed consistent anatomical findings in all three patients who underwent computer assisted medical imagining. All pairs of feet showed abnormal great toes with phalangeal and metatarsal changes, syndactyly of the toes 2 to 5, bony fusions between metatarsals, tarsal coalitions and limitations in shoe fitting. Case 1 was a 2-year-old boy who showed closely approximated calcaneocuboidal joints on CT scan which had not yet reached the stage of definite bony bridging, however, the approximation might suggest the presence of cartilaginous or fibrous fusion. Case 2, a 4-year-old girl presented with a bilateral calcaneocuboidal fusion in the hindfoot. The feet of a 9-year-old boy displayed a synostosis of the hindfoot with talonavicular, talocalcaneal and calcaneocuboidal fusions on CT scan [23].

Carranza-Bencano et al. described a case of a 9-year-old boy with Apert syndrome who presented with calcaneo-cuboid and cuneonavicular fusion in addition to complex syndactyly of all toes and a shortened great toe with medial deviation [24].

Anderson et al. described other anatomical variants of the Apert foot in various case reports. Radiographs revealed additional metatarsal bones in a patient who appeared to have typical feet associated with Apert syndrome upon clinical examination [25]. Another case presented with asymmetrical findings on clinical examination, as there were only four toes present in the right foot compared to the left; however, complex syndactyly was present in both feet. The radiographs, however, showed all metatarsals present with various phalanges of different toes missing in the right foot. Another case presented with similar findings on clinical examination, radiographs, however, showed a synostosis of the proximal phalanx of the third and fourth toes. These case reports strongly reinforce the need for radiographic evaluation of both hands and feet in patients with Apert syndrome in order to determine the underlying bone composition [26].

### Conservative approach

Blauth et al. mentioned orthopaedic arch support insoles of the first and second metatarsal for elevation of pressure points on the third metatarsal head in Apert foot. Furthermore, orthopaedic shoes are an option to help with discomfort caused by lateral weight bearing or great toe deformities [19]. Pedobarography should be used for the identification of locations with elevated pressure in order to make a valid foot care decision regarding support insoles, as Guldemond et al. showed that clinical evaluation overestimated the pressures in the metatarsal region, whereas underestimating the ones in the great toe [27]. No other relevant information concerning further conservative treatment strategies was found in our literature search.

### Treatment recommendations

**Fig. 2:**
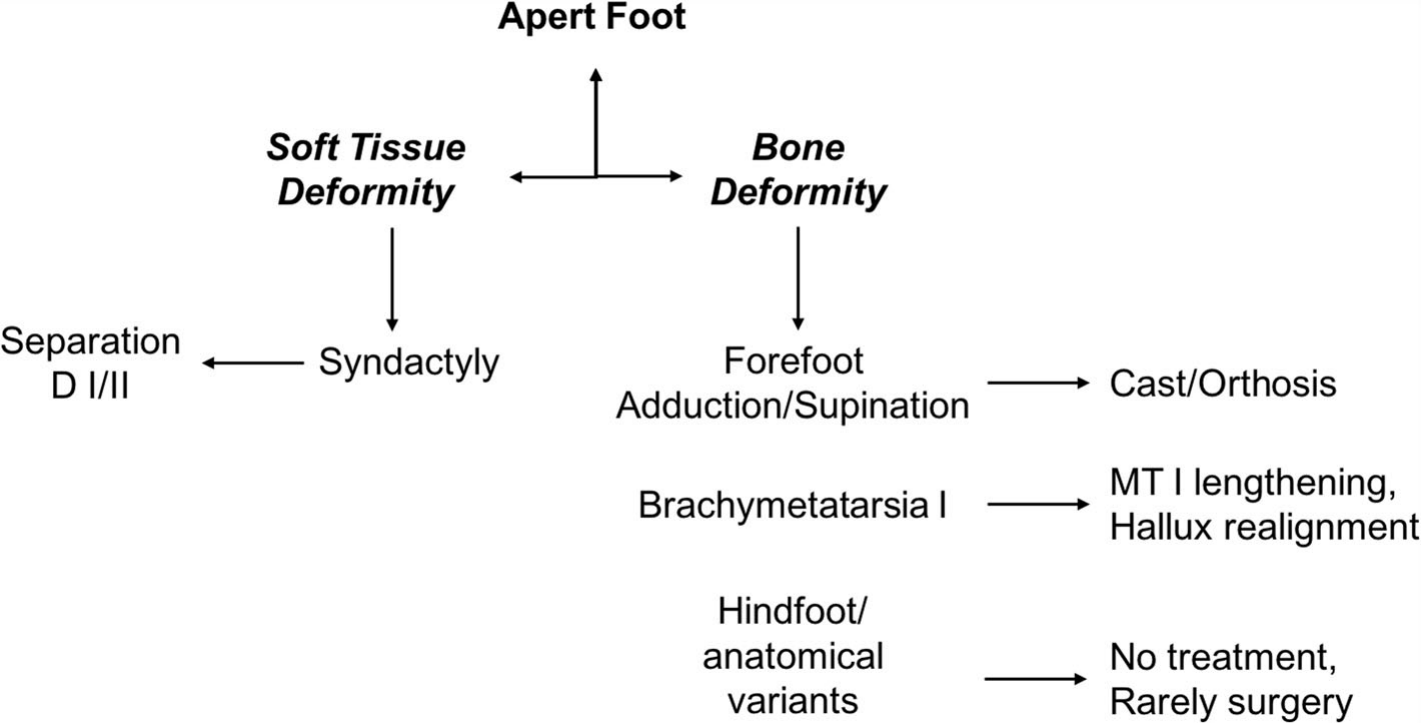
Our treatment proposal is shown. Basically, it has to be distinguished whether soft- or bone deformity is the predominant feature. Thereafter, treatment is aimed to correct soft tissues (e.g. syndactylies) or bone affections using a multitude of conservative or operative means

Children with Apert syndrome should receive early pediatric orthopaedic attention to assess major deformities of the hands and feet. In the latter, bone and soft tissue alterations should be noted and addressed in a staged manner (Fig. 2). Given the fact that craniosynostosis are usually given priority in the first year of life, extremity surgery, if necessary, is usually performed thereafter. The main deformities to be considered for treatment are forefoot adduction deformity, syndactylies, first ray/great toe shortening and malalignment, and hindfoot fusions/bony variants. Based on our personal experience, casts, splints and/or orthoses may be helpful in cases with rigid forefoot adduction and/or supination deformity to achieve a plantigrade foot position. This should be corrected ideally before walking age or latest until the end of the second year. We usually separate syndactylies only if the great toe is involved; we do not see any clinical or true cosmetic advantage in syndactyly separation of the other lesser toes, especially since the rate of local infection and keloid formation is rather high. The children are allowed to wear conventional shoes; however, in pre-school age, thorough clinical, radiographic and pedobarographic assessment is pursued to check for abnormal biomechanics and pressures, and anatomical variants. The shortening of the first ray is usually addressed in pre-school to school-aged children if symptoms and/or markedly elevated plantar pressures are present. However, this surgical intervention, whether performed acutely or in a gradual manner by osteodistraction, can also be performed later if necessary. Pedobarography is an essential tool to help with indication setting for insole prescription, orthopaedic shoe-wear or surgery. Hindfoot deformities and variants such as coalitions or bony fusion may pose a disturbing problem but have to be cautiously addressed on a highly individualized basis. In our experience, many of these findings do not need any specific treatment.

## Conclusions

Due to the multidisciplinary aspect of Apert syndrome, patients undergo multiple surgical corrections primarily focusing on the correction of craniosynostosis and midface retrusion at an early age [28]. Although various surgical options for the correction of the Apert foot have been described in literature, mainly focusing on the correction of syndactyly of the toes, as well as approaching the problem caused of anomalous great toes, no management protocol for the Apert foot has been established thus far. In the past, surgical interventions were reserved only for patients complaining of functional impairment or difficulties when fitting shoes, however, as the life expectancy of patients with Apert syndrome increases these problems may become more present in medical practice. Therefore, research regarding treatment options for the feet is essential in providing optimal care and improvement of quality of life for children born with this rare condition. Pedobarography is capable to highlight areas of increased loading during gait and should be used routinely to monitor development during growth. Based on the clinical findings and symptoms during childhood conservative and/or operative means are followed.

## Data Availability

Data sharing is not applicable to this article as no datasets were generated or analysed during the current study.

## Abbreviations

FGFR: Fibroblast growth factor receptor
CT: Computer tomography

## References

[1] Apert E (1906). De l’acrocéphalosyndactylie. Bull Soc Méd Hôp (Paris).

[2] Tolarova MM, Harris JA, Ordway DE, Vargervik K (1997). Birth prevalence, mutation rate, sex ratio, parents’ age, and ethnicity in Apert syndrome. Am J Med Genet.

[3] Cohen MM, Kreiborg S, Lammer EJ (1992). Birth prevalence study of the Apert syndrome. Am J Med Genet.

[4] Blank CE (1960). Apert’s syndrome (a type of acrocephalosyndactyly)-observations on a British series of thirty-nine cases. Ann Hum Genet.

[5] Glaser RL, Broman KW, Schulman RL, Eskenazi B, Wyrobek AJ, Jabs EW (2003). The paternal-age effect in Apert syndrome is due, in part, to the increased frequency of mutations in sperm. Am J Hum Genet.

[6] Wilkie AO, Slaney SF, Oldridge M (1995). Apert syndrome results from localized mutations of FGFR2 and is allelic with Crouzon syndrome. Nat Genet.

[7] Wenger TL, Hing AV, Evans KN (1993). Apert syndrome. In: Adam MP, Ardinger HH, Pagon RA, et al., eds. GeneReviews®.

[8] Rubin MB, Pirozzi DJ, Heaton CL (1972). Acrocephalosyndactyly. Report of a case, with review of the literature. Am J Med.

[9] Siminel MA, NeamŢu CO, DiŢescu D (2017). Apert syndrome - clinical case. Romanian J Morphol Embryol.

[10] Blauth W, von Törne O (1978). Der “Apert-fuss” [“Apert’s foot” (in acrocephalo-syndactyly) (author’s transl)]. Z Orthop Ihre Grenzgeb.

[11] Cohen MM, Kreiborg S (1995). Hands and feet in the Apert syndrome. Am J Med Genet.

[12] Fearon JA (2003). Treatment of the hands and feet in Apert syndrome: an evolution in management. Plast Reconstr Surg.

[13] Upton J (2003). Treatment of the hands and feet in Apert syndrome: an evolution in management; Jeffrey a. Fearon, M.D. Plast Reconstr Surg.

[14] Rogers M (2002). Nail manifestations of some important genetic disorders in children. Dermatol Ther.

[15] Mah J, Kasser J, Upton J (1991). The foot in Apert syndrome. Clin Plast Surg.

[16] Grayhack JJ, Wedge JH (1991). Anatomy and management of the leg and foot in Apert syndrome. Clin Plast Surg.

[17] Geng X, Shi J, Chen W (2019). Impact of first metatarsal shortening on forefoot loading pattern: a finite element model study. BMC Musculoskelet Disord.

[18] Dell PC, Sheppard JE (1981). Deformities of the great toe in Apert's syndrome. Clin Orthop Relat Res.

[19] Blauth W, Falliner A (1997). Fußkorrekturen beim Apert-Syndrom. Oper Orthop Traumatol.

[20] Calis M, Oznur A, Ekin O, Vargel I (2016). Correction of Brachymetatarsia and medial angulation of the great toe of Apert foot by distraction Osteogenesis: a review of 7 years of experience. J Pediatr Orthop.

[21] Schauerte EW, St-Aubin PM (1966). Progressive synosteosis in Apert’s syndrome (acrocephalosyndactyly), with a description of roentgenographic changes in the feet. Am J Roentgenol Radium Therapy, Nucl Med.

[22] Upton J (1991). Apert syndrome: classification and pathalogic anatomy of limb abnormalities. Clin Plast Surg.

[23] Collins ED, Marsh JL, Vannier MW, Gilula LA (1995). Spatial dysmorphology of the foot in Apert syndrome: three-dimensional computed tomography. Cleft Palate Craniofac J.

[24] Carranza-Bencano A, Gómez-Arroyo JA, Fernández-Torres JJ, Moya CF (2000). Foot deformities associated with Apert syndrome. J Am Podiatr Med Assoc.

[25] Anderson PJ, Smith PJ (1996). Additional metatarsal bones in Apert’s syndrome. The Foot.

[26] Anderson PJ, Smith PJ (1996). Asymmetrical anomalies of the feet in Apert syndrome. The Foot.

[27] Guldemond NA, Leffers P, Nieman FH, Sanders AP, Schaper NC, Walenkamp GH (2006). Testing the proficiency to distinguish locations with elevated plantar pressure within and between professional groups of foot therapists. BMC Musculoskelet Disord.

[28] Allam KA, Wan DC, Khwanngern K (2011). Treatment of apert syndrome: a long-term follow-up study. Plast Reconstr Surg.

